# Nanoimaging granule dynamics and subcellular structures in activated mast cells using soft X-ray tomography

**DOI:** 10.1038/srep34879

**Published:** 2016-10-17

**Authors:** Huan-Yuan Chen, Dapi Meng-Lin Chiang, Zi-Jing Lin, Chia-Chun Hsieh, Gung-Chian Yin, I.-Chun Weng, Peter Guttermann, Stephan Werner, Katja Henzler, Gerd Schneider, Lee-Jene Lai, Fu-Tong Liu

**Affiliations:** 1 Institute of Biomedical Sciences, Academia Sinica, Taipei, Taiwan, ROC; 2 National Synchrotron Radiation Research Center, Taiwan, ROC; 3 Soft Matter and Functional Materials, Helmholtz-Zentrum Berlin, Albert-Einstein-Str. 15, D-12489 Berlin, Germany; 4 Institut für Physik, Humboldt-Universität zu Berlin, Newtonstr. 15, D-12489 Berlin, Germany; 5 Department of Dermatology, UC Davis School of Medicine, Sacramento, CA, USA

## Abstract

Mast cells play an important role in allergic responses. During activation, these cells undergo degranulation, a process by which various kinds of mediators stored in the granules are released. Granule homeostasis in mast cells has mainly been studied by electron microscopy (EM), where the fine structures of subcellular organelles are partially destroyed during sample preparation. Migration and fusion of granules have not been studied in detail in three dimensions (3D) in unmodified samples. Here, we utilized soft X-ray tomography (SXT) coupled with fluorescence microscopy to study the detailed structures of organelles during mast cell activation. We observed granule fission, granule fusion to plasma membranes, and small vesicles budding from granules. We also detected lipid droplets, which became larger and more numerous as mast cells were activated. We observed dramatic morphological changes of mitochondria in activated mast cells and 3D-reconstruction revealed the highly folded cristae inner membrane, features of functionally active mitochondria. We also observed giant vesicles containing granules, mitochondria, and lipid droplets, which we designated as granule-containing vesicles (GCVs) and verified their presence by EM in samples prepared by cryo-substitution, albeit with a less clear morphology. Thus, our studies using SXT provide significant insights into mast cell activation at the organelle level.

Mast cells are important immune effector cells that contribute to the allergic response and have various immunomodulatory functions. Their progenitor cells arise from the bone marrow, pass through the blood vessels before they migrate to tissues, and then differentiate into mature mast cells. Most abundantly distributed at sites near the host-environment interfaces, such as the skin and mucosal tissues, mast cells are well suited for the first line of defense against invading pathogens or other environmental insults^1^. These cells are characterized by their high amounts of electron-dense secretory granules that fill a large proportion of their cytoplasm^2^. These granules contain a plethora of preformed and pre-activated immunomodulatory compounds, including lysosomal enzymes, biogenic amines, such as histamine, and proteoglycans^3^. Upon activation, mast cells undergo degranulation, where these preformed compounds are rapidly released into the extracellular environment. Activated mast cells also release some newly synthesized mediators, including leukotrienes, prostaglandins, cytokines, chemokines, and growth factors. Through these released compounds, mast cells can modulate various physiological and pathological events. Importantly, some of the released mediators from the granules can cause allergic responses, such as those occurring in asthma and allergic rhinitis. Thus, studies of how mast cell granule contents are released are vital to our understanding of allergic diseases.

Our current understanding of degranulation is based on ultrastructural studies using transmission electron microscopy (TEM) and immunofluorescence studies using confocal microscopy. A complete understanding of the process of mast cell degranulation requires a thorough understanding of the morphological processes underlying this complex process. A number of morphological studies have been performed, largely by TEM. These various studies have led to a number of apparent contradictions. For example, there are currently two competing models for degranulation: anaphylactic degranulation in which the fusion of secretory vesicles leads to formation of secretory channels and piecemeal exocytosis in which small granules bud off the larger granules and individually fuse with the plasma membrane to release their contents. There has also been debate about the role of mitochondria and the requirement for energy in the degranulation process.

One reason for this variety of different conclusions could be that TEM studies are usually limited to analyzing just a few thin sections from 3D specimens. This makes it difficult to identify relatively rare structures in a cell. In addition, the TEM preparation procedure can introduce artifacts due to chemical fixation, dehydration or physical sectioning, such as distorted or disorganized organelles, altered membrane continuity, or appearance of empty space in the cytoplasm^4^,^5^, and these artifacts can vary depending on the particular procedures used, leading to different interpretations from different groups.

To generate a more comprehensive picture of the degranulation process, we have applied the emerging technique of soft X-ray tomography (SXT), which is capable of imaging ultrastructure of hydrated intact cells in three dimension (3D) and is complementary to TEM in observing the structures of organelles. SXT covers the energy in the water window that is between the K-absorption edge of carbon and oxygen (284–543 eV). Coincidentally, the main components in biological specimens are carbon, nitrogen, and oxygen. Therefore, SXT images can be generated from the different absorption coefficients between the biological specimen and water, which is a naturally occurring contrast; thus, there is no need to stain or dehydrate the specimens. Moreover, the penetration depth of photons in the soft X-ray energy range is more than that of electrons in biological samples. SXT produces images of biological specimens that represent a section of up to 10 μm thick, so there is no need to section the cells before imaging^4^,^6^. SXT can reveal the 3D structure of biological specimens to around 35 nm of half-pitch resolution in their near native state^7^.

Here, we employed SXT to study organelles of activated rat basophil leukemia (RBL) cells, a model for mast cells^8^, at near-native morphology. We observed and reconstructed the 3D structure of granules and their dynamics. We also observed giant vesicles that contain well discernable organelles, including granules, mitochondria, and lipid bodies. This study demonstrates the advantages of SXT in preserving organelle structures and biomembrane integrity. SXT can thus shed light on mast cell degranulation mechanisms, including the near-native subcellular activities of organelles.

## Results

### Using SXT to visualize mast cell degranulation

**Figure 1:**
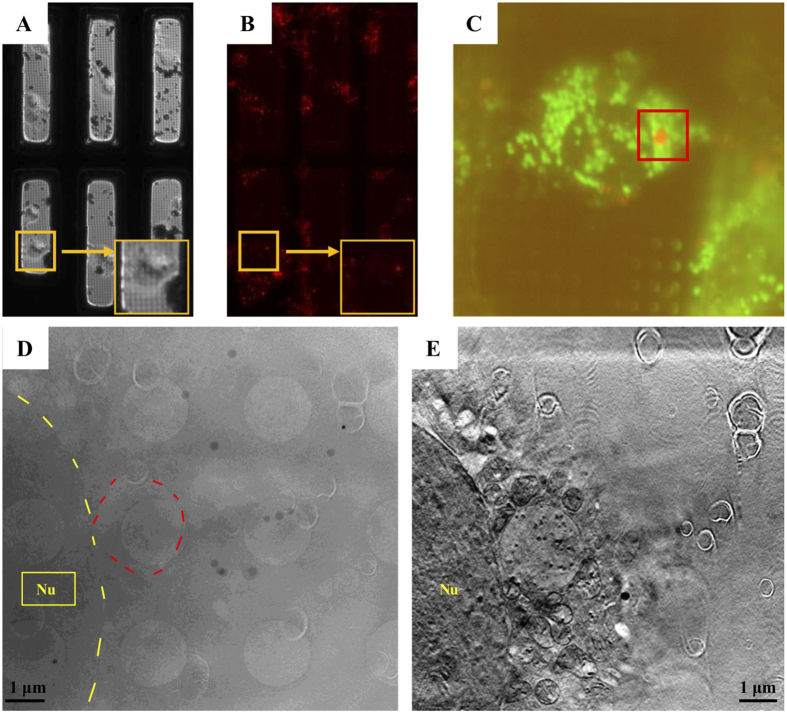
Sample preparation for cryo-soft X-ray tomography (SXT). RBL-2H3 cells were grown on gold grids coated with Quantifoil holey-carbon films. The cells were prelabeled with fluorescent dye and frozen with a plunge freezer in liquid ethane. (**A,B**) Cryo-brightfield and cryo-fluorescence images of cells were captured with a 10x objective lens on the cryo-fluorescence screening system at −150 °C for screening of the samples. (**C**) Fluorescent images of the cryo-cells were captured with a 100x objective lens in the vacuum chamber to search for granular structures at U41-SGM beamline TXM endstation, BESSY II. Mitochondria (green) and granules (red) are visible. (**D**) An image captured by SXT was coordinated with the fluorescent dye stained granule (circle labeled) shown in (**C**). (**E**) A reconstructed SXT image by IMOD to show morphological structures of the organelles. Nu: nucleus.

RBL cells can be sensitized by IgE via their cell surface high-affinity IgE receptor (FcεRI). When these surface IgE molecules bind to specific antigens, the cells are activated to release various substances mentioned above. Anti-dinitrophenyl (DNP) IgE-sensitized RBL cells grown on gold grids were stimulated by antigen [DNP-bovine serum albumin (BSA)] via FcεRI-mediated activation, rapidly frozen, and subsequently imaged using correlative cry-fluorescence and SXT (Fig. 1A,B) to determine the location of granules (Fig. 1C), followed by SXT (Fig. 1D). The raw data were reconstructed using the IMOD software (Fig. 1E). The reconstructed images were coordinated with the fluorescent images to identify the granules.

### Granular morphology during mast cell degranulation

**Figure 2:**
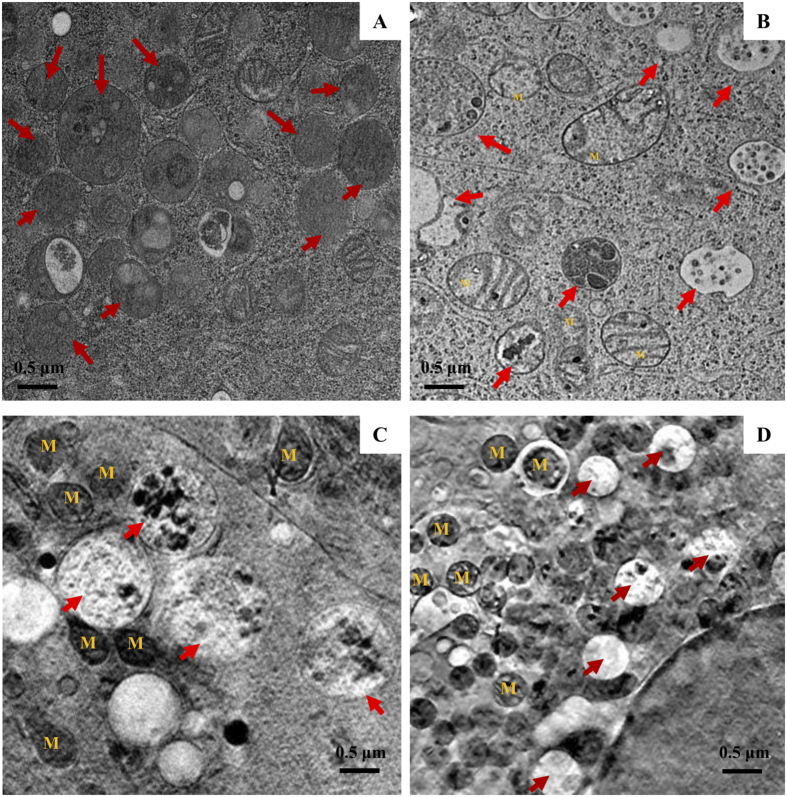
Granular morphology during mast cell degranulation. RBL-2H3 cells were sensitized with anti-DNP IgE and then stimulated with or without DNP-BSA for 30 min. (**A,B**) Transmission electron microscopy (TEM) images of cells either not stimulated (**A**) or stimulated with DNP-BSA (**B**) followed by ultrathin section from Spurrs’ resin embedded cells. (**C,D**) Reconstructed images of SXT of cells non-stimulated (**C**) or stimulated with DNP-BSA (**D**). Arrows indicate granules and M indicates mitochondrion.

To facilitate the identification of the subcellular structure of mast cells during degranulation, we conducted freeze-substitution TEM in parallel with SXT to visualize the organelles. The TEM results showed that IgE-sensitized RBL cells without stimulation contained highly electron-dense secretory granules (Fig. 2A). After antigen stimulation, granules became electron-lucent (Fig. 2B). The three dimensional SXT images demonstrated that IgE-sensitized RBL cells without stimulation contained highly carbon-dense secretory granules (Supplementary video S1), which was correlated with TEM results. Morphological analysis for granule sizes did not show significant size changes upon cell activation. This is the first observation of 3D images inside the granules. In the SXT images before antigen stimulation, some inclusion particles (dense cores) with diameters of 50 to 100 nm were observed inside the granules. They contained high densities of carbon, which forms natural contrast due to photon absorption at 510 eV without any staining (Fig. 2C and Supplementary video S2). After 30 min of antigen stimulation, we observed release of the inclusions from the granules (Fig. 2D, Supplementary video S3). From the 3D images, we determined the percentages of inclusions released from the granules to be 37.36 and 15.21 (N = 12) with the standard deviations of 13.23 and 9.08, for nonstimulated and stimulated cells, respectively.

### Granule dynamics in mast cell degranulation

**Figure 3:**
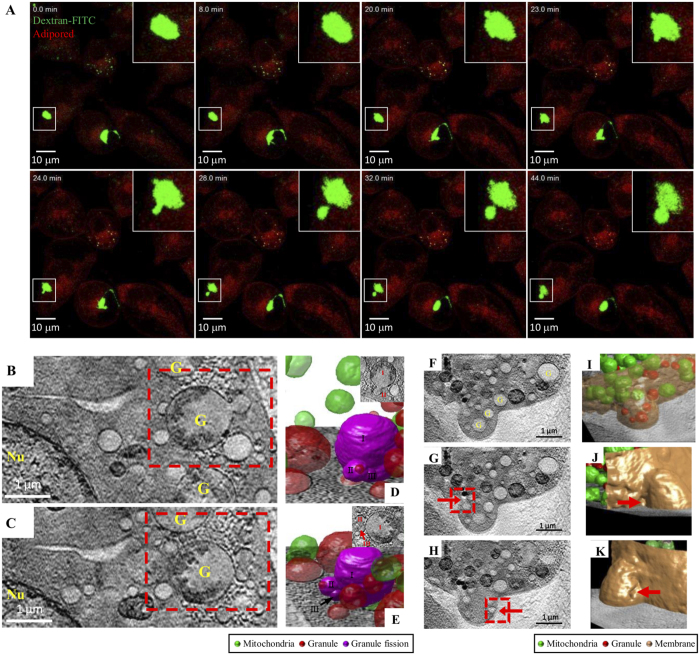
Granule dynamics in mast cell degranulation. Cells were sensitized with anti-DNP IgE and then stimulated with DNP-BSA at different time points. (**A**) Fluorescence microscopy image of cells pre-stained with dextran-FITC (green) and adipored (red). Scale bars represent 10 microns. Cells were sensitized with anti-DNP IgE, and then stimulated with DNP-BSA for 3 min. (**D,E,I–K**) 3D structure reconstructions from SXT. (**B,C,F–H**) Segment images of cells in SXT reconstructions. (**D,E**) A magnification from the rectangle in panel (**B,C**) in different orientation. Arrow indicates a cavity in the membrane protrusion region. Granules: red; mitochondria: green; granule fission: violet; cell membrane: dark yellow. Scale bars represent 1 micron. Nu: nuclear; G: granule.

Mast cell degranulation involves a number of membrane events, including fusion and fission of granules and granule-plasma membrane fusion. Granules fuse with the plasma membrane before they release their contents into the surrounding environment. We employed confocal microscopy and SXT to study granule change and membrane dynamics. Fluorescence-labeled dextran was used to target secretory granules in RBL cells through endocytosis^9^. From serial time-lapse confocal images of granules in dextran-FITC pulsed RBL cells, we observed small vesicles budding and separating from larger granules when the cells were activated (Fig. 3A). This type of images, to our knowledge, has not been reported previously.

By SXT, we also observed granule fission in RBL cells at an early time point (3 min) after antigen stimulation. We noted a large granule apparently being divided into three smaller granules, two of them being close to the plasma membrane (Fig. 3B,C and Supplementary video S4). The 3D reconstructed images from a segment of Fig. 3B,C are shown at different angles (Fig. 3D,E).

Supplementary data 4 shows Z stack images after reconstruction and the 3D structure of the organelles inside the cells. The small granules might have fused with the plasma membrane and formed curvatures. Supplementary TEM images show that granule mediators were released near the membrane cavities (Supplementary Fig. S5). We also observed cell membrane protrusions in activated cells (Fig. 3F–H). A 3D image of the membrane protrusion is shown in Fig. 3I, which contains several granules, and some of them appear to be moving away from the cell membrane. Cavities in this membrane protrusion region are observable (Fig. 3G,H, boxed region, Fig. 3J,K). This protruding membrane structure provides evidence for vesicles releasing their contents from degranulating cells.

### Mitochondrial translocation and cristae remodeling in degranulating mast cells

**Figure 4:**
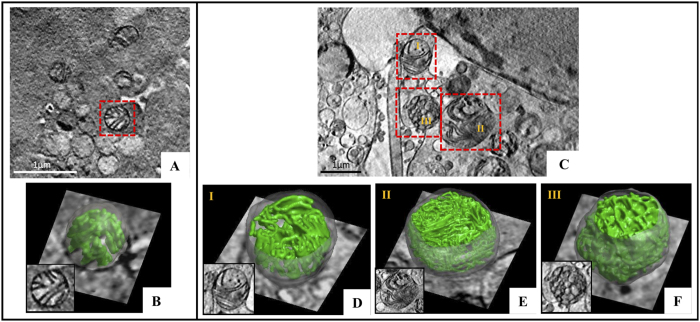
Mitochondrial changes in mast cell degranulation. Cells were sensitized with DNP-IgE and then stimulated with DNP-BSA at different time points. (**A,C**) Segment images of cells from SXT reconstructions before and after 30 min of activation. (**B,D–F**) 3D reconstructed structures from SXT in the rectangle area of (**A**) and I, II, III of (**C**).

Mast cell degranulation requires intracellular calcium and metabolic energy^10^. Mitochondria are the primary energy-generating organelles in eukaryotic cells, and mitochondrial dynamics allow them to participate in various complex cellular processes and contribute to cell proliferation, death, and differentiation^11^. Mitochondria can translocate to subcellular regions that require high metabolic activity in various cell types^12^,^13^,^14^. In peritoneal mast cells undergoing intense degranulation, a large number of mitochondria was found to localize to the cell surface close to the sites of exocytosis^15^. We observed the detailed morphology and structure of mitochondria during mast cell activation by SXT. In antigen-stimulated RBL cells, the cristae structure of the mitochondria was increased and features of cristae remodeling were observed (Fig. 4). Reconstructed 3D images showed that before mast cell activation, the cristae of mitochondrial inner membrane were loose with little indentation (Fig. 4B, Supplementary video S6), but after activation, they became densely connected with increased amount of inner membrane folding and indentation (Fig. 4C–F, Supplementary video S7). We classified mitochondria into the following three types based on the condensation of their cristae: type I: lamellar morphology (Fig. 4D); type II: a mixture of lamellar morphology and hole shape (Fig. 4E); and type III: hole shape (Fig. 4F). Notably, type II and III are activation specific because they were only observed in stimulated cells. Morphometric analysis of mitochondria based on the condensation state of their cristae indicates that the average percentages of mitochondria type I, II, III (Fig. 4D–F) are 70, 20, and 10%, respectively.

### Accumulation of lipid bodies in degranulating mast cells

**Figure 5:**
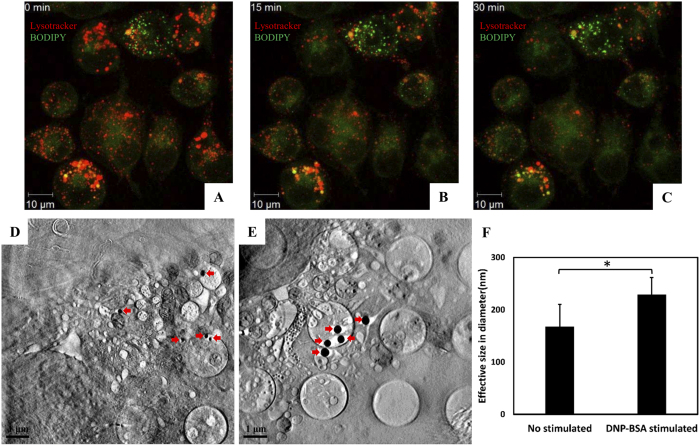
Lipid body accumulation in mast cell degranulation. Cells were sensitized with anti-DNP IgE and then stimulated with DNP-BSA at different time points. (**A–C**) Fluorescence microscopy images of cells stained with Lysotracker-DNP 99 (red) and BODYPY (green) after 0, 15, and 30 min. Scale bars represent 10 microns. (**D,E**) Sectioned reconstructed SXT images after cells were sensitized with anti-DNP IgE and then stimulated for 30 min with DNP-BSA. Lipid bodies are indicated by red arrows. Scale bars represent 1 micron. (**F**) The comparison of the mean effective size of lipid bodies between cells with and without DNP-BSA stimulation. Bars represent the mean of 50 lipid bodies calculated from eight tomographic images from unstimulated cells and seven tomographic images from stimulated cells; error bars represent standard deviation (s.d.); *denotes p < 0.05.

Lipid bodies are formed during mast cell maturation^16^ and are found, for example, in insulin-treated RBL cells^16^. They are characterized as organelles that are distinct from secretory granules^17^. We compared lipid bodies in mast cells by fluorescence microscopy and SXT. To identify lipid body accumulation during mast cell degranulation, we stained antigen-activated mast cells with BODIPY 495/503 and tracked the granules with Lysotracker DNP-99. By time-lapse confocal live imaging, we found that lipid droplets were increased in number and accumulated around granules in activated cells (Fig. 5A–C). By SXT, we also observed lipid bodies in non-stimulated (Fig. 5D) and antigen-stimulated RBL cells (Fig. 5E). The sizes of lipid bodies were larger in antigen-stimulated cells than in unstimulated cells (Fig. 5D,E), which we confirmed by quantitative analysis (Fig. 5F).

### Granule-containing vesicles are revealed by SXT and freeze substitution TEM

**Figure 6:**
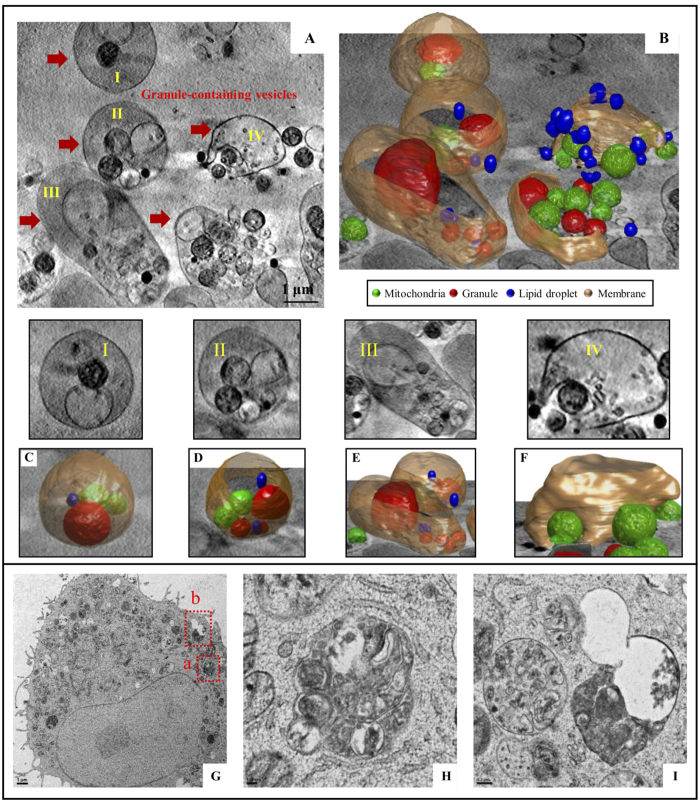
Granule-containing vesicles in mast cell degranulation by SXT and freeze substitution TEM. Cells were sensitized with anti-DNP IgE and then stimulated with DNP-BSA for 30 min. (**A**) A segment image of a cell from SXT reconstructions. (**B**) A 3D image of Granule-containing vesicle from (**A**). (**C–F**) Magnification of I-IV in (**A**). (**G**) A TEM image of a section from freeze-substituted and Spurrs’ resin embedded cells. (**H,I**) Magnification of Granule-containing vesicles in part a and b from panel (**G**), respectively. Granule: red; mitochondria: green; GCV membrane: dark yellow; lipid bodies: blue. Scale bars represent 1 micron.

Intracellular granules fuse and form large vesicles upon mast cell stimulation^18^. The structures of fused granules have been studied by traditional TEM methods with chemical fixation^19^. By SXT, we observed multiple large vesicles with heterogeneous constituents in RBL cells after 25 to 45 min of antigen stimulation, but not in the early stages of stimulation or in non-stimulated cells (Fig. 6A and Supplementary video S8). We named these large vesicles “granule-containing vesicles” (GCVs) and further characterized their structures and contents by high magnification 3D reconstruction (Fig. 6B–F and Supplementary videos S9 and S10). By analyzing the image contrasts caused by different carbon densities, we identified the contents as empty granules, dense granules, lipid bodies, and mitochondria. Our image results also showed mitochondria engulfed by GCVs (Fig. 6A-IV,F), indicating that the formation of GCVs may result from the incorporation of organelles. By freeze-substitution TEM, we also identified GCV-like vesicles in RBL cells after 30 min of antigen stimulation (Fig. 6G–I).

## Discussion

Mast cell granules are electron dense secretory vesicles constituted of preformed compounds, including histamine, protease, and glycosaminoglycan^3^. In response to environmental stimuli, mast cells undergo activation and release granule contents through exocytosis. The mechanism of mast cell degranulation has been extensively studied and is generally defined by two models. One model, “anaphylactic degranulation”, involves extensive fusion of secretory granules with each other to form a “degranulation channel”, followed by fusion of the granules with the cell membrane^3^,^20^. The other model called “piecemeal exocytosis” is a process in which small vesicles bud off parental granules without granule-granule or granule-plasma fusion, resulting in loss of granule content^3^,^18^,^21^. In our current study, we observed granule budding by using SXT imaging (Fig. 3B–E), which was also supported by immunofluorescence analysis (Fig. 3A). This fits better what is described for the “piecemeal exocytosis” process. However, granule fusion to plasma membrane was observed, indicating the possible existence of anaphylactic degranulation. Thus, these two degranulation processes may be occurring simultaneously. We also observed in activated mast cells granule fusion occurring in conjunction with plasma membrane dynamics that resembles filopodia formation (Fig. 3G,H,J,K). This suggests that when being activated, mast cells also receive cues to undergo migration.

The reconstructed 3D images of the granules, along with other organelles, such as mitochondria, allowed us to visualize the subcellular spatial localization of organelles. We observed that the mitochondria undergo cristae remodeling when mast cells are activated. Abnormal mitochondrial cristae have been observed in mast cells under pathological conditions such as in diabetic rats^22^,^23^. Cristae remodeling is closely related to mitochondria function such as energy biogenesis^24^. For example, the shape of mitochondria cristae affects respiratory chain assembly and the efficiency of energy generation^25^. By SXT imaging, we reconstructed 3D images of mitochondria cristae and showed the differences in cristae before and after mast cell activation, indicating that mast cell degranulation is likely energy-dependent. Indeed, higher energy consumption is coupled with mitochondria translocation from perinuclear distribution to the cell surface and a rapid increase in intracellular calcium levels^26^. The near-native 3D images of mitochondria generated by SXT allowed us to further investigate the structure-function relationship of mitochondria under specific conditions, including one related to the allergic responses. We conclude that degranulation is energy dependent based on the presence of mitochondria and in particular on the cristae morphology. Here the excellent preservation of membranes by SXT is critical to allow visualization of the morphological changes in the mitochondrial cristae.

Lipid bodies have been studied as the site of arachidonic acid metabolism as well as eicosanoid synthesis and storage^27^,^28^. The 3D morphology of lipid bodies has been studied by conventional TEM, immunogold EM, and electron tomography^29^. In human mast cells, lipid bodies have no correlation with mast cell degranulation in terms of volume and morphology^16^,^17^. However, we found an increase in lipid body volume during mast cell activation. Whether this is due to a difference in the animal species needs to be further investigated. Our study also showed the association of lipid bodies with mitochondria and granules (Fig. 5A–C and 6B). Indeed, lipid bodies have been reported to be associated with mitochondria through perilipin 5, a lipid droplet-associated protein^30^, and SNAP-23^31^, the phosphorylated form of which is involved in mast cell degranulation^32^. Thus, these structures may contact granules through SNAP-23.

Our SXT images revealed compact structures of degranulation channels that we designated granule-containing vesicles (GCVs). Structures similar to these have been reported in resting mouse bone marrow-derived mast cells (BMMCs) by TEM and are reminiscent of multivesicular bodies (MVBs)^33^. In addition, exosome-containing secretory granules have been observed in RBL cells^34^,^35^,^36^. Importantly, however, compared to previously reported structures, our SXT images show that GCVs contain granules and other organelles, including mitochondria and lipid bodies. These structures might represent autophagosomes that are formed because of organelle engulfment by some isolation membranes or lysosomes. Autophagy has indeed be reported to be induced in activated mast cells and plays a role in degranulation^33^. However, we believe formation of GCVs is an integral part of the degranulation process. Multiple granules are known to fuse with each other before reaching the plasma membrane. This can result in more efficient release of granule components since granules farther from the plasma membrane could release their content by fusion with other granules closer to the plasma membrane without having to migrate toward the plasma membrane.

Alternatively, multiple granules might fuse with the plasma membrane simultaneously, followed by GCV formation during plasma membrane reconstruction. It is likely the organelles included in GCVs are exocytosed once the latter fuse with the plasma membrane. This is consistent with the previous observation that mitochondria are released from activated mast cells *in vitro* and *in vivo*^15^. Our findings suggest that lipid bodies may also be released into the extracellular space through GCV-associated degranulation. Thus, formation of GCVs resulting from granule-granule or granule-plasma membrane fusion may be considered a part of the anaphylactic type of degranulation.

Our studies and others using SXT demonstrate several advantages of this technology compared with those of other techniques such as 3D EM tomography: 1) cells can be imaged in their near native state - they do not have to be extensively processed, as required for electron microscopy; 2) structures can be observed using a natural contrast rather than by using stains; 3) it is possible to obtain three-dimensional information without collecting serial sections of 10-μm thickness in the water window energy. However, the main disadvantage of SXT compared with the 3D EM tomography technique is spatial resolution. Visualizing the fusion process of the granule membrane with the plasma membrane is out of reach for SXT; only 3D EM tomography can provide such an enhanced spatial resolution. Our studies demonstrate that SXT is well suited to study the structural dynamics of intracellular organelles/structures and obtain a 3D view in nearly their native form, upon change in cellular activation status.

## Methods

### Cell culture

The rat basophilic leukemia (RBL-2H3) cell line was grown in Dulbecco’s modified Eagle’s medium (DMEM) supplemented with 20% fetal bovine serum (FBS), 100 U/ml penicillin and 100 μg/ml streptomycin. Cells were cultured at 37 °C in 5% CO_2_.

### Chemical and reagents

LysoTracker Red DND-99 and MitoTracker Green FM were purchased from Molecular Probes/Invitrogen. FITC-dextran (150 kDa) were purchased from Sigma.

### Degranulation assays

RBL-2H3 cells (2.5 × 10^4^ cells per well) were cultured in 96 well plates overnight at 37 °C and sensitized with anti-2,4-dinitrophenyl (DNP) IgE^37^ (0.5 μg/ml) overnight. IgE sensitized cells were washed twice in Tyrode’s buffer (135 mM NaCl, 5 mM KCl, 5.6 mM glucose, 1.8 mM CaCl_2_, 1 mM MgCl_2_, 20 mM HEPES and 0.5 mg/ml BSA (pH 7.4)) and stimulated with 100 ng/ml multivalent DNP-bovine serum albumin (DNP-BSA) for 45 min at 37 °C. β-Hexosaminidase release was measured as previously described^38^.

### Sample preparation for microscopy

RBL-2H3 cells were cultured in 35 mm coverslip dishes in media containing 0.5 μg/ml anti-DNP IgE in the presence or absence of FITC–dextran (1 mg/ml). IgE-sensitized cells were further treated with adipored (14 μl per 1 ml culture media), LysoTracker Red DND-99 (1 μM) and MitoTracker Green FM (500 nM) (or BODIPY 493/503(1 μM)) for 30 min staining at 37 °C. After staining, cells were washed twice with Tyrode’s buffer. Cells were stimulated with 100 ng/ml DNP-BSA and time-lapse images were taken over one hour by the Zeiss LSM780 confocal microscope system.

### Soft X-ray tomography

RBL cells were stimulated via IgE receptor (FcεRI)-mediated activation and subjected to SXT. Cells were grown on gold grids coated with holy-carbon films and prelabeled with a fluorescent dye that highlights granules and mitochondria. The grids were frozen immediately by plunge freezing in liquid ethane. The samples were imaged by the cryo-fluorescence screening system to ensure the suitability of the samples, before image acquisition by SXT (Fig. 1A,B). In U41-SGM beamline HZB TXM endstation at BESSY II, the cells on the grids were first placed in the vacuum chamber to capture fluorescent images with the incorporated light microscope and determine the location of granules (Fig. 1C). RBL-2H3 cells were grown on specialized HZB-2 Au-grids coated with Holey Carbon Films (Quantifoil) and sensitized with anti-DNP IgE overnight. After sensitization, the grids with RBL-2H3 cells were stained with LysoTracker Red DND-99 (1 μM)/MitoTracker Green FM (500 nM) for pre-screening with visible light fluorescent microscopy. Cells were stimulated with DNP-BSA (100 ng/ml) for different amounts of time. Afterward, the grids were fast frozen. First, the grids were placed in the FEI Vitrobot freezing system and gold particles (100 nm or 250 nm) were added. After 3 sec of incubation and blotting to remove the water, the grids were quickly dropped into liquid ethane in the Vitrobot freezing system. The plunge frozen samples were loaded into the cryo-correlative screening system (Linkam Scientific Instruments) and fluorescence microscope (Zeiss Axioscope A1) for cryo-sample post-screening at National Synchrotron Radiation Research Center, Taiwan (NSRRC). The ice condition was selected before transferring the cryo-sample to BESSY II, Helmholtz Zentrum Berlin for SXT. The cryo-samples were maintained under cryogenic conditions during the shipping.

SXT tilt series were obtained by using the full-field transmission x-ray microscope at U41-SGM beamline. Prior to soft-X-ray imaging, the LysoTracker Red DND-99 and MitoTracker Green FM signals were screened by beamline equipped fluorescent microscope (online fluorescent microscope) to locate the cells of interest. Once selected, the cell was imaged by soft X-ray at photon energy of 510 eV with 25 nm zone plate objective. Tilt images of selected cell were collected by rotating the sample with the increment of 1 degree. Background corrected tilt series were then proceeded to reconstruct the volumes of stack images by freely available program IMOD (http://bio3d.colorado.edu/imod) using filtered back projection (FBP) and iterative reconstruction algorithms. Furthermore, segmentation and 3D presentation of the final volumes of the targeted cell were done by MATLAB^®^ and Avizo.

### Freeze-substitution TEM

Following the same sample preparation process of SXT, the frozen samples were maintained under cryogenic conditions. After SXT processing, the samples were transferred into the Reichert–Jung AFS freeze-substitution unit (Leica, Vienna, Austria) and the temperature was increased from −140 °C to −90 °C. Pure acetone with 0.1% (w/v) uranyl acetate and 1% OsO_4_ was added to the samples for 60 h at −90 °C. Samples were brought from −90 °C to −20 °C and finally to room temperature and then embedded in Spurrs’ Resin. Capsules were polymerized for 1 day at 60 °C. The polymerized capsules were cut by ultrathin sectioning and transferred to copper grids. The grids were stained with saturated uranyl acetate for 30 min and lead citrate for 5 min. The stained sections were visualized on a J FEG-TEM, FEI Tecnai G2 TF20 S-TWIN microscope (operating at 100 kV).

## Additional Information

**How to cite this article**: Chen, H.-Y. *et al.* Nanoimaging granule dynamics and subcellular structures in activated mast cells using soft X-ray tomography. *Sci. Rep.* **6**, 34879; doi: 10.1038/srep34879 (2016).

## Supplementary Material

Supplementary Information

Supplementary Video S1

Supplementary Video S2

Supplementary Video S3

Supplementary Video S4

Supplementary Video S5

Supplementary Video S6

Supplementary Video S7

Supplementary Video S8

Supplementary Video S9

Supplementary Video S10
