# Corrigendum: Nanoimaging granule dynamics and subcellular structures in activated mast cells using soft X-ray tomography

**DOI:** 10.1038/srep40458

**Published:** 2017-01-19

**Authors:** Huan-Yuan Chen, Dapi Meng-Lin Chiang, Zi-Jing Lin, Chia-Chun Hsieh, Gung-Chian Yin, I.-Chun Weng, Peter Guttmann, Stephan Werner, Katja Henzler, Gerd Schneider, Lee-Jene Lai, Fu-Tong Liu

Scientific Reports 6: Article number: 34879; 10.1038/srep34879 published online: 10 17 2016; updated: 01 19 2017.

The original version of this Article contained an error in the spelling of the author Peter Guttmann, which was incorrectly given as Peter Guttermann.

This has now been corrected in the PDF and HTML versions of the Article.

