# Supplementary material for: Nanoimaging granule dynamics and subcellular structures in activated mast cells using soft X-ray tomography
Source: Sci Rep. 2016 Oct 17;6:34879. doi: 10.1038/srep34879 (PMC5066221; doi:10.1038/srep34879)

Nanoimaging granule dynamics and subcellular structures in activated mast cells using soft X-ray tomography

Huan-Yuan Chen[1], Meng-Lin Chiang[1], Zi-Jing Lin[2], Chia-Chun Hsieh[2], Gung-Chian Yin[2], I-Chun Weng[1], Peter Guttermann[3], Stephan Werner[3] and Katja Henzler[3], Gerd Schneider[3,4], Lee-Jene Lai[2], Fu-Tong Liu[1,5,*]

Supplementary Information

Supplementary video S1: Reconstructed soft X-ray tomography 3D video of granules in non-activating mast cells

RBL-2H3 cells were grown on gold grids coated with Quantifoil holey-carbon films, sensitized with anti-DNP IgE and then kept for 30 min without stimulation. The cells were prelabeled with fluorescent dye and frozen with a plunge freezer in liquid ethane. Images of the cryo-cells were captured at the U41-SGM HZB beamline TXM endstation, BESSY II. The video is a reconstructed SXT image by IMOD and MATLAB software to show morphological 3D structures of granules.

Supplementary video S2: Granule morphology in non-activating mast cell

The samples were prepared as in Supplementary video S1. This is the stack images of a SXT image reconstructed by IMOD software.

Supplementary video S3: Granule morphology in activating mast cell

The samples were prepared as in Supplementary video S1, except IgE-pulsed cells were stimulated with DNP-BSA for 30 min.

Supplementary video S4: Granule dynamics in mast cell degranulation by SXT

The samples were prepared as in Supplementary video S3, except IgE-pulsed cells were stimulated with DNP-BSA for 3 min.

Supplementary Fig. S5: Granule cavity formation in mast cell degranulation from freeze-substitution TEM

The samples were prepared as in Supplementary video S3, except the images are freeze-substitution TEM images of the cryo-cells captured by FEI Tecnai G2 TF20 S-TWIN microscopy.

Supplementary video S6: Reconstructed SXT 3D video of mitochondria in non-activating mast cells

The samples were prepared as in Supplementary video 5B.

Supplementary video S7: Reconstructed SXT 3D video of mitochondria in activating mast cells

The procedure is the same with Supplementary video 5F.

Supplementary video S8: Granule-containing vesicles in mast cell degranulation from SXT

The video corresponds to Fig. 7A.

Supplementary video S9: Reconstructed soft X-ray tomography 3D video of Granule-containing vesicles in mast cell degranulation from soft X-ray tomography

The video corresponds to Fig. 7C.

Supplementary video S10: Reconstructed soft X-ray tomography 3D video of Granule-containing vesicles in mast cell degranulation from soft X-ray tomography

The video corresponds to Fig. 7F.

Supplementary video S11: Time-lapse images of granule dynamics upon mast cell activation Anti-DNP IgE-sensitized RBL Cells were pre-stained with lysotracker (red) and bodipy (green) and then stimulated with DNP-BSA for 45 min. Scale bars represent 5 microns.

Supplementary Fig. S5: Granule cavity formation in mast cell degranulation from freeze-substitution TEM

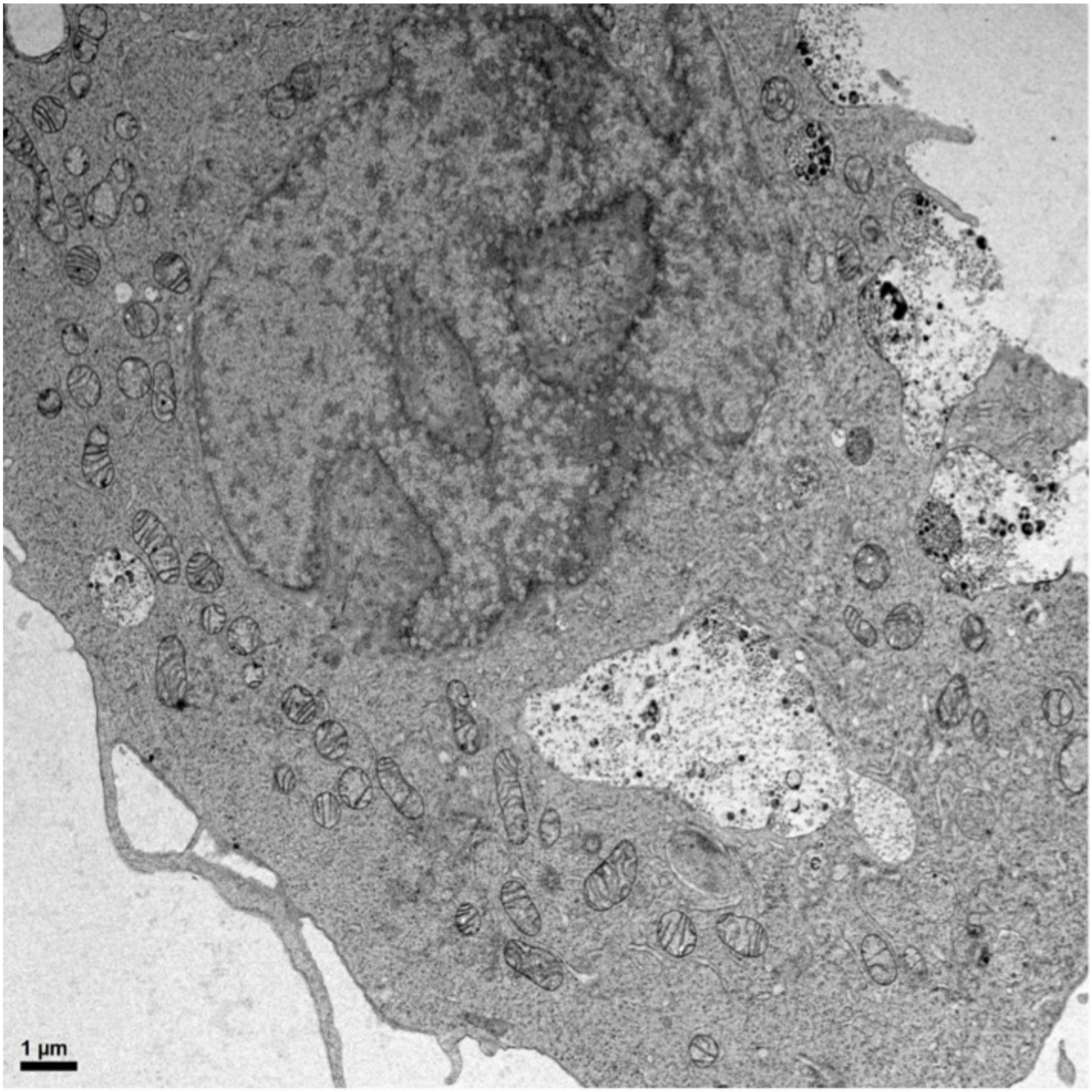

Supplement: Supplementary Information [file srep34879-s1.pdf]
